# Extra virgin olive oil improves synaptic activity, short‐term plasticity, memory, and neuropathology in a tauopathy model

**DOI:** 10.1111/acel.13076

**Published:** 2019-11-24

**Authors:** Elisabetta Lauretti, Miroslav Nenov, Ozlem Dincer, Luigi Iuliano, Domenico Praticò

**Affiliations:** ^1^ Alzheimer’s Center at Temple Lewis Katz School of Medicine Temple University Philadelphia Pennsylvania; ^2^ Department of Medico‐Surgical Sciences and Biotechnology Sapienza University of Rome Latina Italy; ^3^ UOC Internal Medicine ICOT University Hospital Sapienza University of Rome Latina Italy

**Keywords:** extra virgin olive oil, hTau, memory, synaptic plasticity, tau pathology, tauopathy

## Abstract

In recent years, increasing evidence has accumulated supporting the health benefits of extra virgin olive oil (EVOO). Previous studies showed that EVOO supplementation improves Alzheimer's disease (AD)‐like amyloidotic phenotype of transgenic mice. However, while much attention has been focused on EVOO‐mediated modulation of Aβ processing, its direct influence on tau metabolism in vivo and synaptic function is still poorly characterized. In this study, we investigated the effect of chronic supplementation of EVOO on the phenotype of a relevant mouse model of tauopathy, human transgenic tau mice (hTau). Starting at 6 months of age, hTau mice were fed chow diet supplemented with EVOO or vehicle for additional 6 months, and then the effect on their phenotype was assessed. At the end of the treatment, compared with control mice receiving EVOO displayed improved memory and cognition which was associated with increased basal synaptic activity and short‐term plasticity. This effect was accompanied by an upregulation of complexin 1, a key presynaptic protein. Moreover, EVOO treatment resulted in a significant reduction of tau oligomers and phosphorylated tau at specific epitopes. Our findings demonstrate that EVOO directly improves synaptic activity, short‐term plasticity, and memory while decreasing tau neuropathology in the hTau mice. These results strengthen the healthy benefits of EVOO and further support the therapeutic potential of this natural product not only for AD but also for primary tauopathies.

## Graphical abstract

Chronic administration of extra‐virgin olive oil increases synaptic activity and short‐term plasticity, improves memory, and reduces tau neuropathology.

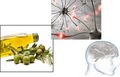

## 1 INTRODUCTION

Alzheimer's disease (AD) and related tauopathies are neurodegenerative disorders characterized by the presence of highly phosphorylated and misfolded forms of the microtubule‐associated tau protein, which accumulate as neurofibrillary or gliofibrillary tangles in the human brain (Di Meco, 2019). Currently, the mechanisms and cellular pathways leading to aberrant tau phosphorylation and progressive accumulation are not fully understood (Arendt, 2016) and no therapy is available to cure or halt the progression of these pathologies. The most striking symptoms common to all these diseases are the loss of memory and deficits in cognitive function. In fact, the brain of tauopathy patients reveals severe synaptic degeneration, neuronal dysfunction, and ultimately neuronal loss. Studies in animal models of the disease have shown that the early stages of tauopathy are characterized by alterations in both pre‐ and postsynaptic turnover rates, reduction of synaptic strength and dendritic spine size, and decrease in spontaneous and evoked cortical neuronal activity (Busche et al., 2019; Jackson et al., 2017).

Remarkably, tau pathology strictly correlates with the severity of dementia (Arriagada, Growdon, Hedley‐Whyte, & Hyman, 1992; Riley, Snowdon, & Markesbery, 2002). Tau toxicity has been mostly attributed to its loss of function as a microtubule stabilizer, which leads to impaired neuronal transport, altered mitochondria function, and synaptic transmission (Lasagna‐Reeves et al., 2012). Tau phosphorylation and other post‐translational modifications promote tau self‐aggregation initially into oligomers and eventually into neurofibrillary tangles. Tau oligomers are often found at both pre‐ and postsynaptic sites in AD, supporting the notion that they contribute to synaptic transmission dysfunction (Guerrero‐Muñoz, Gerson, & Castillo‐Carranza, 2015; Tai et al., 2012).

Recently, several lines of evidence have demonstrated the health effects of extra virgin olive oil (EVOO) and have suggested that its consumption ameliorates age‐related memory decline and decrease the risk of AD onset (Farr et al., 2012; Feart, Samieri, Alles, & Barberger‐Gateau, 2013; Pitozzi et al., 2010). While most of the experimental studies have been focusing on the ability of EVOO to enhance Aβ clearance and reduce its deposition (Abuznait, Qosa, Busnena, El Sayed, & Kaddoumi, 2013; Qosa et al., 2015), only fewer research groups have been investigating the tau‐modifying ability of EVOO. On this regard, studies reported that some natural phenolic derivatives from olives, as well as oleocanthal, can prevent tau fibrillization in vitro (Daccache et al., 2011; Monti, Margarucci, Tosco, Riccio, & Casapullo, 2011). More recently, we have shown that chronic administration of an EVOO‐rich diet modulates positively several aspects of the AD‐like pathology in a transgenic mouse model with plaques and tangles, the 3×Tg mice (Lauretti Iuliano, & Praticò, 2017; Lauretti, Li, Di Meco, & Praticò, 2017). However, since this model manifests both aspects of the AD pathological phenotype, whether EVOO can modulate tau neuropathology independently from Aβ remains to be fully investigated. This is an important biological question with relevance not only for AD but also for primary tauopathies, such as progressive supranuclear palsy and Pick's disease, which are characterized by the presence of only tau pathology. With this goal in mind, in the present paper we evaluated the effect of chronic EVOO administration in tau transgenic mice (hTau) in which the mouse tau gene is replaced by normal human tau (Andorfer et al., 2003).

## 2 RESULTS

### 2.1 EVOO‐rich diet ameliorates learning and memory in hTau mice

To determine the effect of EVOO‐rich diet on learning ability and memory, animals were tested at the end of the study, after 6 months of diet (at age of 12 months) in three different paradigms: Y‐maze, Morris water maze, and novel object recognition.

**Figure 1:**
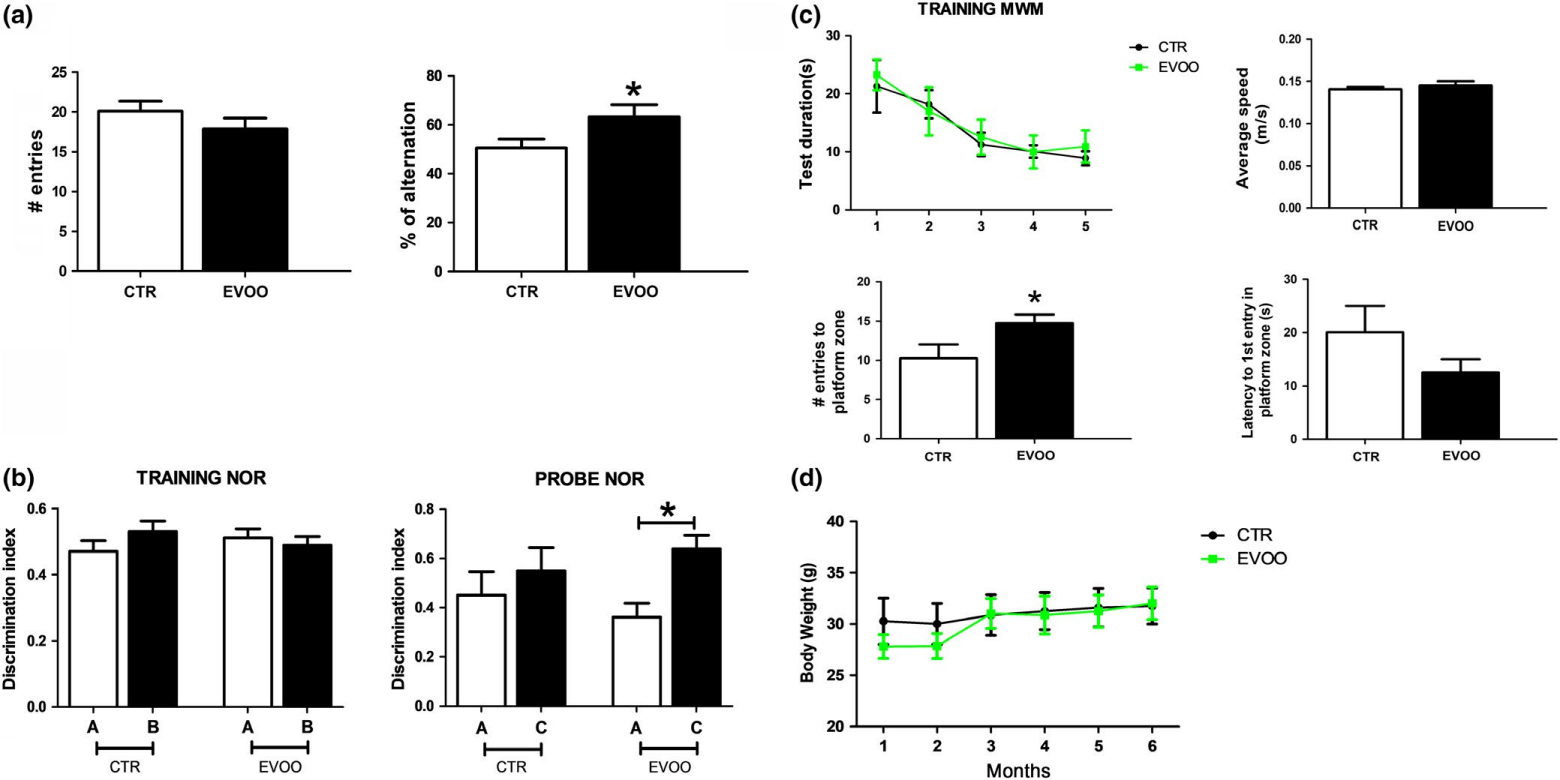
Chronic administration of EVOO‐rich diet ameliorates behavioral impairments in hTau mice. Starting at 6 months of age, hTau mice were randomized to receive regular chow diet (CTR) or diet enriched with EVOO (EVOO) until they were 12 months old. (a) Number of entries and percentage of alternation in the Y‐maze paradigm (*p < .05). (b) Discrimination index in the training and probe phase of novel object recognition task for both groups (CTR *n* = 8, EVOO, *n* = 9) (*p < .05). (c) Training phase of Morris water maze (MWM) as measured by latency to reach the platform zone over five consecutive days, number of entries to platform zone, latency to initial platform crossing, and swimming speed for the two groups in the probe trial (CTR *n* = 8, EVOO, *n* = 9) (*p < .05). (d) Total body weight of CTR (*n* = 8) and EVOO (*n* = 9) mice from the beginning until the end of the study. Values represent mean ± *SEM*

As shown in Figure 1a, in the Y‐maze, when compared with the control group, mice receiving the EVOO‐rich diet showed a significant increase in the percentage of alternation but no difference in the number of entries, indicating that the while EVOO diet improved working memory it did not affect their normal locomotor activity. Next, mice underwent the novel object recognition task. During the training phase, mice did not display any preference for one side of the cage or for one of the two identical objects. However, in the probe trial, while the control group showed no preference for the novel object, which demonstrates lack of recall or loss of memory, the EVOO group spent significantly more time with the novel object suggesting memory improvement (Figure 1b). Finally, animals were tested in the Morris water maze paradigm. All mice in each group were able to visually find the platform (data not shown) and to reach the training criterion within 5 days (Figure 1c). No differences were found during the training session and in their swimming speed between the two groups (Figure 1c). During the probe test, we observed that compared with controls, mice receiving the EVOO‐rich diet manifested a significant increase of the number of entries in the platform zone (Figure 1c).

Compared with regular diet, the EVOO‐rich diet had no effect on total body weight of the mice, since we did not observe any significant difference in this parameter between the two groups throughout the study (Figure 1d). No significant differences in the results of any of the behavioral tests implemented were observed when males and females were analyzed separately (not shown).

### 2.2 Effect of EVOO on hippocampal synaptic activity in hTau mice

**Figure 2:**
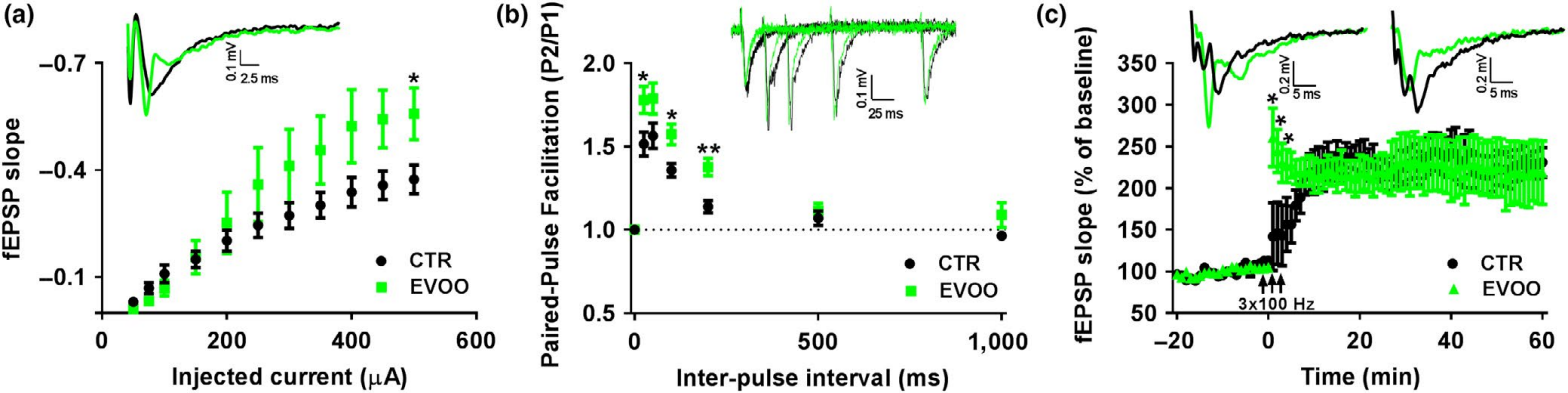
Effect of EVOO‐rich diet on basal synaptic activity, short‐term and long‐term plasticity in hTau mice. (a) Input–output curve of fEPSP slope vs injected current in hTau (CTR) and hTau on EVOO diet (EVOO) mice. The upper inset shows representative voltage traces of fEPSP induced with stimulus intensity of 500 µA. (b) Time dependence of paired‐pulse facilitation of fEPSP slope as a ratio of fEPSP slope at pulse 1 to fEPSP slope at pulse 2 for different time points. The upper inset shows representative voltage traces of paired‐pulse fEPSP at 25 msec, 50 msec, 100 msec, and 200 msec inter‐pulse intervals. (c) Long‐term potentiation induced with 3 consecutive pulses of high‐frequency stimulation of 100 Hz and 1 s inter‐pulse interval. The fEPSP slope values are presented as a percentage of response after LTP induction to averaged response of baseline. The upper insets show representative voltage traces of fEPSP 1 min after LTP induction (left inset) and 60 min after LTP induction (right inset). (CTR = 7, EVOO = 7 number of slices each). *p < .05, **p < .01

To elucidate changes in synaptic activity that may underlie the beneficial effect of EVOO‐rich diet on the observed hippocampal‐associated behavioral performance, we studied basal synaptic activity, STP, and LTP in the CA1 area of hippocampus of control and EVOO‐rich diet treated mice. To this end, we stimulated the Schaffer collaterals as an input and recorded fEPSP as an output response from *stratum radiatum* dendrites of the CA1 area to probe the activity of CA3‐CA1 synapses. As show in Figure 2a, we found an enhanced basal synaptic activity as the analysis of input–output curve revealed significant increase in fEPSP slope induced by administration of current at high intensity in the EVOO diet treated mice when compared to controls. Next, we studied the effect of EVOO diet on short‐term plasticity by measuring the time dependency of paired‐pulse facilitation (PPF). For this parameter, the ratio of fEPSP slope at pulse 2 to fEPSP slope at pulse 1 was measured at different inter‐pulse intervals. Compared with controls, we observed that slices from EVOO group had a significant increase in the paired‐pulse ratio at a broad range of inter‐pulse time intervals, thus exerting the potentiating effect on PPF (Figure 2b). Finally, we tested the effect of EVOO‐rich diet on LTP using the high‐frequency stimulation (HFS) protocol. In this test, we found that right after the LTP induction with HFS, slices from the controls had a significant failure in post‐tetanus potentiation, which is another form of short‐term plasticity, which in contrast was significantly ameliorated in the EVOO‐treated group (Figure 2c). However, there was no significant difference at the mid‐late phase of LTP recording between the two groups (Figure 2c).

### 2.3 Effect of EVOO on hippocampal pre‐ and postsynaptic proteins in hTau mice

**Figure 3:**
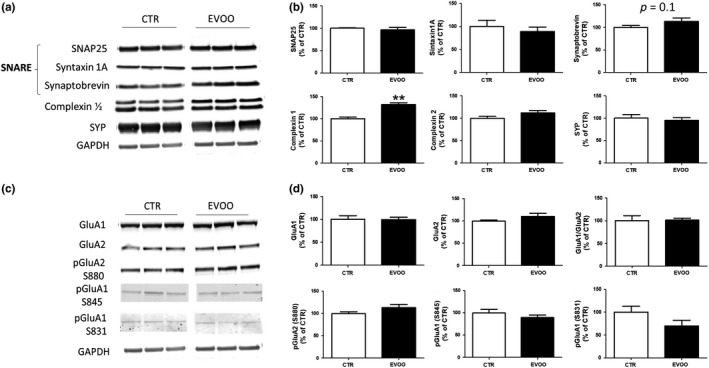
Effect of chronic administration of EVOO‐rich diet on hippocampal pre‐ and postsynaptic protein expression in hTau mice. (a) Representative Western blot analyses for SNAP25, syntaxin 1A, synaptobrevin, complexin 1/2, and synaptophysin (SYP) in hippocampus homogenates from CTR and EVOO mice. (b) Densitometric analyses of the immunoreactivities to the antibodies shown in panel A (**p < .01; *n* = 6 per group)**.** (c) Representative Western blot analyses for the AMPAR subunit glutamate receptor 1 (GluR1), AMPAR subunit glutamate receptor 2 (GluR2), and their phosphorylated forms, pGluR1 (S831), pGluR1 (S845), and pGluR2 (S880), in hippocampus homogenates from CTR and EVOO mice. (d) Densitometric analyses of the immunoreactivities to the antibodies shown in panel A (*n* = 6 per group)**.** Values are mean ± *SEM*

To elucidate the mechanisms involved in the modulation of basal hippocampal synaptic activity and STP by EVOO‐rich diet, next we measured levels of pre‐ and postsynaptic proteins including SNARE complex, complexin 1/2, and synaptophysin that are critical for a proper function of the presynaptic vesicle release machinery, as well as postsynaptic AMPA receptor subunits phosphorylation and protein levels which are responsible for basal synaptic activity (Neeliyath et al., 2012; Trimbuch & Rosenmund, 2016). Among all the tested proteins, we found that only complexin‐1 levels were significantly increased in the EVOO‐treated hTau mice when compared with controls (Figure 3a,b). In addition, the same group had a trend toward an increase in the steady‐state levels of synaptobrevin (Figure 3b), and a decrease in the levels of AMPA receptor subunit GluA1 phosphorylated at serine 831 (Figure 3c, d). However, in both cases the differences did not reach statistical significance.

### 2.4 Effect of EVOO supplementation on tau neuropathology of hTau mice

**Figure 4:**
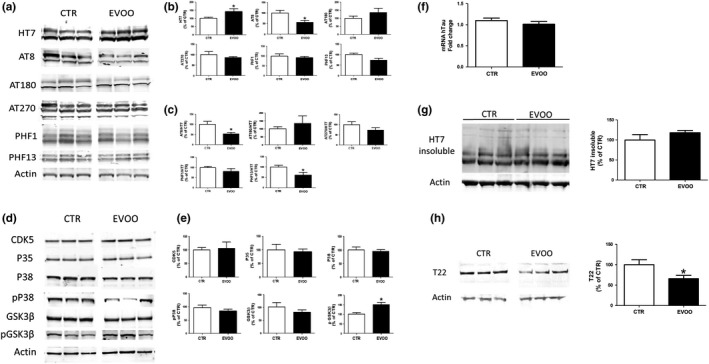
EVOO‐rich diet regulates tau phosphorylation and tau oligomers accumulation in the brain of hTau mice. (a) Representative Western blot analyses for soluble tau (HT7) and phosphorylated tau at residues S202/T205 (AT8), T181 (AT270), T231 (AT180), S396/S404 (PHF1), and S396 (PHF13) in brain cortex homogenates from control (CTR) and EVOO‐treated mice (EVOO). (b) Densitometric analyses of the immunoreactivities to the antibodies shown in panel A (*p < .05, *n* = 6 per group). (c) Densitometric analyses of the phospho‐tau/total tau ratio for the different epitope immunoreactivities shown in panel A (*p < .05, *n* = 6 per group). (d) Representative Western blot analyses for cyclin‐dependent kinase CDK5, P35, glycogen synthase kinase (GSK3β, p‐GSK3β), P38 MAPK kinase, and pP38 MAPK protein levels in brain cortex homogenates from CTR and EVOO mice. (e) Densitometric analyses of the immunoreactivities to the antibodies from panel D (*p < .05) (f) MAPT gene expression level in brain cortex homogenates from CTR and EVOO mice. The mRNA expression levels of tau (MAPT) were determined using qRT–PCR analysis (*n* = 6 per group). (g) Representative Western blot analyses for formic acid‐soluble tau (HT7) in brain cortex homogenates from CTR and EVOO mice and densitometry analysis (*n* = 6 per group). (h) Representative Western blot analyses for tau oligomers as recognized by T22 antibody in brain cortex homogenates from CTR and EVOO mice and densitometry analysis (*p < .05, *n* = 6 per group). Values are means ± *SEM*

To assess the effect of EVOO‐rich diet on tau phosphorylation and pathology, we measured levels of total soluble tau protein and its phosphorylated isoforms in brain cortex from both groups. As shown in Figure 4a, b, we found that while levels of total soluble tau were significantly increased, tau phosphorylated at Ser202/Thr205, as recognized by the AT8 antibody, was significantly reduced when EVOO‐rich diet group was compared to control. By contrast, no significant changes were observed between the two groups of mice when tau phosphorylated at Thr231, Thr181, Ser396/Ser404, and Ser396 as recognized by the antibodies AT180, AT270, PHF1, and PHF13 were assessed, respectively (Figure 4a,b). However, when we analyzed the phospho‐tau/total tau ratio for the different epitopes we found a significant reduction also for tau phosphorylation at the specific Ser396 epitope, as recognized by PHF13 antibody (Figure 4c).

To identify potential mechanisms that may be responsible for the observed changes in tau phosphorylation in the EVOO group, we measured the levels of the principal kinases involved in these tau post‐translational modifications. When comparing control and EVOO mice, immunoblot analysis showed no change for total glycogen synthase kinase 3‐β (GSK3‐β) but a significant increase in the inhibitory Ser9 phosphorylation of GSK3‐β (Figure 4d,e). By contrast, no differences between the two groups were observed when steady‐state levels of CDK5, P35, and total or phosphorylated P38 were assessed (Figure 4d,e). Since EVOO‐rich diet treated mice displayed higher total soluble tau protein levels, to determine whether this was resulting from an effect on transcription of the MAPT gene, we assessed tau mRNA levels. As shown in Figure 4f, RT‐qPCR analysis revealed no significant changes in tau mRNA levels between the two groups.

To fully characterize tau pathology, we also looked at two additional toxic forms of tau protein: the formic acid‐soluble tau aggregates and the soluble aggregates known as tau oligomers, as recognized by tau oligomer‐specific antibody T22 (Lasagna‐Reeves et al., 2012). As shown in Figure 4g and h, although there was no change in the formic acid‐soluble form of tau, EVOO‐treated mice presented a significant reduction in tau oligomers levels when compared with controls.

### 2.5 Effect of EVOO on synaptic proteins and neuroinflammation

**Figure 5:**
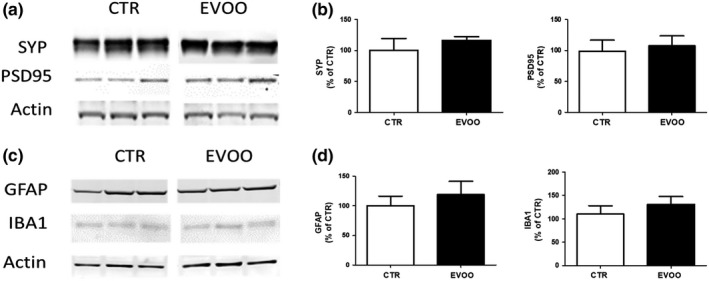
Effect of chronic administration of EVOO‐rich diet on synaptic integrity and neuroinflammation. (a) Representative Western blot analyses of synaptophysin (SYP) and postsynaptic density protein 95 (PSD95) in brain cortex homogenates of control (CTR) and EVOO‐treated (EVOO) hTau mice (*n* = 6 per group). (b) Densitometric analyses of the immunoreactivities to the antibodies shown in the previous panel. (c) Representative Western blot analyses of GFAP and IBA1 in brain cortex homogenates of CTR and EVOO mice (*n* = 6 per group). (d) Densitometric analyses of the immunoreactivities to the antibodies shown in the previous panel. Values represent mean ± *SEM*

Since tau phosphorylation and tau oligomers are known to target synapse, next we investigated whether overall synaptic integrity of the hTau mice was affected by the EVOO‐rich diet. To this end, we assayed the steady‐state levels of synaptophysin (SYP) indices of presynaptic integrity and the postsynaptic density protein 95 (PSD‐95) in cortices of the two groups of mice. As shown in Figure 5a, b, no significant differences were observed between the two groups when steady‐state levels of these proteins were assessed.

Give that EVOO is known to be rich in polyphenols, potent antioxidant, and anti‐inflammatory agents (Bayram et al., 2012; Rigacci, Stefani, Segura‐Carretero, & Gómez Caravaca, 2016), we also investigated two well‐established markers of neuroinflammation: IBA1, marker of microglia activation, and GFAP, marker of astrocytes activation. As shown in Figure 5c and d under our experimental conditions, compared with controls the cellular inflammatory response in the central nervous system of these mice was not influenced by the dietary regimen.

## 3 DISCUSSION

The findings presented in this paper show that chronic administration of a diet enriched in EVOO results in a significant improvement of working memory, spatial and learning memory, amelioration of hippocampal basal synaptic activity and short‐term plasticity, and a significant reduction in tau neuropathology in a relevant mouse model of human primary tauopathy.

In recent years, evidence has been accumulating in support of the beneficial effects of dietary consumption of EVOO, a key component of the Mediterranean diet, in terms of brain health (Farr et al., 2012; Feart et al., 2013; Pitozzi et al., 2010). In particular, EVOO‐rich diet has been associated with lower risk of AD, mild cognitive impairment, and dementia (Peterson & Philippou, 2016). The PREDIMED‐NAVARRA randomized clinical trial involving a cohort of 268 subjects (74.1 ± 5.7 years old) showed that long‐term intervention with an EVOO‐rich Mediterranean diet (6.5 years) results in better cognitive performances, especially across fluency and memory tasks, and less MCI incidence as compared to controls (Martinez‐Lapiscina et al., 2013). Moreover, looking specifically at progression of well‐established AD biomarkers, including β‐amyloid deposition, glucose metabolism, and neuronal loss, a more recent longitudinal study confirmed that, in middle‐aged adults, strong adherence to the Mediterranean diet (MeDi) provide significant protection against AD (Valls‐Pedret et al., 2015). Consistently, another randomized clinical trial showed that in an older population Mediterranean diet supplemented with EVOO (1 L/week) was associated with improved cognitive function after 4.1 years follow‐up (Berti et al., 2018).

Studies in animal models of the disease have revealed that this natural product positively influences many aspects of the AD pathology, including memory, neuroinflammation, and amyloidosis (Abuznait et al., 2013; Qosa et al., 2015). Although much attention has been devoted to the latter aspect of the AD phenotype, the ability of EVOO and its derivatives to potentially modulate also phosphorylation and aggregation of tau protein so far has been evaluated only using in vitro systems (Daccache et al., 2011; Li et al., 2009). Our group recently explored the effect of EVOO on the triple transgenic mouse model of AD, which is known to develop Aβ plaques and tau tangles. Notably, in this model besides an improvement of cognition we found a significant reduction of Aβ peptides and tau phosphorylation (Lauretti, Iuliano, et al., 2017; Lauretti, Li, et al., 2017). However, since in this model Aβ can drive tau phosphorylation and subsequent pathology, the results obtained in that study would not address the important question of whether the observed effect of EVOO on tau was dependent or independent from the effect on Aβ. This is an important biological question with relevance not only for AD pathophysiology, but also for the pathogenesis of other dementing disorders called primary tauopathies, such as progressive supranuclear palsy and Pick's disease, which are characterized by the exclusive presence of tau pathology.

Thus, in order to dissect the effect of EVOO on tau pathology and on tau‐dependent synaptic dysfunction, in the current paper, we used a pure tauopathy mouse model, which expresses wild‐type human tau protein. This model is characterized by age‐dependent impairment in cognitive function, synaptic activity as well as progressive loss in pre‐ and postsynaptic integrity, and for these reasons represents an ideal model system to investigate the biological effect of chronic administration of EVOO (Alldred, Duff, & Ginsberg, 2012; Polydoro, Acker, Duff, Castillo, & Davies, 2009).

At the end of the treatment, compared with hTau mice kept on a regular diet, the hTau EVOO group displayed better working memory, as assessed by the percentage of spontaneous alternations in the Y‐maze paradigm. Moreover, chronic consumption of EVOO ameliorated spatial learning and recognition memory ability as demonstrated by increased number of entries to the platform zone and higher discrimination index, respectively, for the Morris water maze and the novel object recognition test. Importantly, the diet did not alter general motor function of these animals since no significant differences were observed between the two groups when the number of entries in each arm of the Y‐maze and the swim speed in the Morris water maze was assessed.

To prove that these benefits were associated with changes in hippocampal synaptic activity and plasticity, we assessed synaptic function by using field potential recordings in the Schaffer collateral (CA3)‐CA1 synapses. Importantly, this analysis revealed an elevation in basal synaptic activity and short‐term plasticity in the EVOO‐treated mice providing the mechanism and the biological substrate for the observed beneficial effect on cognition. Additionally, we found that EVOO‐rich diet affected in a positive manner also two forms of short‐term plasticity: increased paired‐pulse facilitation and restoration of post‐tetanic potentiation. Taken together, these data support the novel idea that EVOO directly exerts a positive effect on synaptic activity and short‐term plasticity specifically at the presynaptic level.

Interestingly, our data are in agreement with studies showing that oleuropein, one of the main components of EVOO, facilitates hippocampal synaptic activity and long‐term plasticity (Pu et al., 2005), whereas in the CA1 area of hippocampus of TgCRND8 mice it restores post‐tetanic potentiation and long‐term potentiation (Luccarini et al., 2015).

To further elucidate potential mechanisms by which EVOO modulated synaptic activity in our mice, we tested for different proteins considered key pre‐ and postsynaptic components for hippocampal synaptic function. Among them, we found that complexin 1 (CPLX1) levels were significantly higher in the EVOO‐treated group than in controls. CPLX1 is a presynaptic protein known as SNARE complex‐binding protein which plays an important role in vesicle release promoting synchronization of Ca^2+^ triggered vesicle fusion and inhibiting spontaneous vesicles release (Lai et al., 2014). Downregulation of CPLX1 is associated with impaired basal synaptic strength and short‐term plasticity (Chang et al., 2015), and more recently decreased CPLX1 levels have been suggested as a significant risk factor associated with AD and Parkinson's disease (Lahut et al., 2017; Ramos‐Miguel et al., 2017). Our results further support the importance of CPLX1 for the maintenance of basal synaptic activity and short‐term plasticity and provide new evidence on the involvement of this protein in neurodegenerative disorders associated with tau pathology. In addition, they highlight CPLX1 as a novel target of EVOO at synaptic level, and represent the first demonstration that chronic consumption of EVOO by driving upregulation of CPLX1 facilitates synaptic function in the hippocampus.

Since progressive accumulation of phosphorylated tau is another feature of the phenotype in hTau mice, next we assessed the effect of chronic administration of EVOO on this aspect of tau neuropathology. In agreement with previous findings, we saw that the EVOO‐rich diet resulted in a significant reduction in tau phosphorylation at specific epitopes. Mechanistically, this effect was secondary to the increase in the inhibitory phosphorylation at Ser9 of GSK3β kinase, which was found in the EVOO group when compared with controls. However, we observed that EVOO‐treated mice had a significant elevation of total soluble tau protein levels, which was not secondary to a similar change at the mRNA levels. Since tau hyper‐phosphorylation precedes oligomers and tangles formation, we also looked at these two additional forms of toxic tau under our experimental conditions and found that although the insoluble tau fraction was not changed, EVOO‐treated mice had a significant decrease in the levels of tau oligomers.

These findings are highly relevant to the current debate in the field on whether the soluble forms of tau rather than the insoluble ones are indeed more prone to alter synaptic and dentritic normal properties (Tracy & Gan, 2018). Interestingly, growing evidence indicates that the presence of tau oligomers at the synapses induces abnormal synaptic plasticity, reduces the number of dendritic spines and impairs LTP (Guerrero‐Muñoz et al., 2015). Thus, the observed increase in nonphosphorylated soluble tau together with the reduction in tau phosphorylated and oligomeric tau species would strongly suggest that neurons from EVOO‐treated mice had more readily available functional tau protein compared to control, which then correlated with better neuronal health and synaptic communication found in the same experimental group.

In conclusion, our paper demonstrates that in tau transgenic mice chronic administration of EVOO‐rich diet results in an amelioration of working memory, spatial learning, basal synaptic activity, and short‐term plasticity together with a significant reduction in the amount of phosphorylated tau and tau oligomers. Collectively, our findings represent a significant step forward in the in vivo research effort on the biological effect of EVOO on tau pathology and synaptic function. They provide strong preclinical evidence in support of the novel concept that EVOO should be considered as a potential and viable multi‐targeting agent not only for AD but also for primary tauopathies.

## 4 EXPERIMENTAL PROCEDURES

### 4.1 Animals and treatment

All animal procedures were approved by the Institutional Animal Care and Usage Committee, in accordance with the US National Institutes of Health guidelines. The hTau mouse model designed to express all human tau isoforms in absence of endogenous mouse tau was used in this study (Andorfer et al., 2003). Animals were kept in a pathogen‐free environment, on a 12‐hr light/dark cycle and fed a normal chow and water ad libitum. Male and female mice were used throughout the studies and starting at 6 months of age randomized into two groups: one, control (CTR, *n* = 6 females, *n* = 9 males), fed with standard chow diet (PicoLab Rodent Diet 20, Labdiet, St. Louis, MO), and the other fed the same chow diet supplemented with EVOO (50 mg/Kg diet) (*n* = 7 females, *n* = 9 males). The two diets were always matched for calories.

The EVOO used in our study was from the Apulia region of Italy, and its chemical analysis revealed the following: total polyphenols (253 mg/kg), α‐tocopherol (381 mg/kg), and γ‐tocopherol (23 mg/kg). Additionally, fatty acid analysis was performed by gas chromatography as described previously (Corradini et al., 2014) and the results are shown in Table 1. Fresh diet was provided every other day. When animals reached 12 months of age, they underwent behavioral testing as described below and then euthanized. After perfusion brains were immediately removed, gently rinsed in cold 0.9% phosphate‐buffered saline and immediately dissected and stored at −80°C for biochemistry.

**Table 1:** Fatty acid composition of the EVOO used in the study

Fatty acid	Name	Percentage
C14:0	Myristic acid	0.017
C16:0	Palmitic acid	10.157
C18:0	Stearic acid	1.724
C20:0	Arachidic acid	0.287
C22:0	Behenic acid	n.d.
C24:0	Lignoceric acid	n.d.
C26:0	Cerotic acid	n.d.
C14:1	Myristoleic acid	n.d.
C16:1	Palmitoleic acid	0.545
C18:1n‐7c	Vaccenic acid	26.334
C18:1n‐9c	Oleic acid	50.301
C20:1n‐9	Gondolic acid	0.775
C22:1n‐9	Erucic acid	n.d.
C24:1n‐9	Nervonic acid	0.003
C18:2n‐6c	Linoleic acid	7.131
C18:3n‐6c	γ‐Linoleic acid	n.d.
C20:2n‐6	Eicosadienoic acid	0.003
C20:3n‐6	Di‐homo‐γ‐linolenic acid	0.097
C20:4n‐6	Arachidonic acid	0
C22:2n‐6	Docosadienoic acid	2.308
C22:4n‐6	Adrenic acid	n.d.
C22:5n‐6	Osbond acid	n.d.
C18:3n‐3	α‐linolenic acid	n.d.
C20:3n‐3	Eicosatrienoic acid	n.d.
C20:3n‐5	Eicosapentaenoic acid (EPA)	0.042
C22:3n‐5	Docosapentaenoic acid (DPA)	0.171
C22:6n‐3	Docosahexaenoic acid (DHA)	0.007
C20:3n‐9	Mead acid	0.013
C18:1n‐9t	Elaidic acid	n.d.
C18:2n‐6t	Linoleadic acid	0.002
C16:1n‐7t	Palmiteleaidic acid	0
C16:1n‐9c	Cis‐7‐hexadecenoic	0.070
C18:2n‐7t	9, 11 all‐trans linoleic acid	0.011

Abbreviation: n.d., not detectable (below the detection limit)

### 4.2 Behavioral tests

All the animals were handled for at least 3–4 consecutive days before testing, and all tests were conducted by an experimenter blind to the conditions.

### 4.3 Y‐maze

The Y‐maze apparatus consisted of 3 arms, 32 cm (long) 610 cm (wide) with 26‐cm walls (San Diego Instruments, San Diego, CA). As previously described (Lauretti, Iuliano, et al., 2017; Li, Barrero, Merali, & Praticò, 2017), testing was always performed in the same room and at the same time to ensure environmental consistency. After introduction to the center of the Y‐maze, the animals freely explored the three arms for 5 min and the sequence and total number of arms entered was video‐recorded to calculate number of entries and percentage of alternation. Entry into an arm was considered valid if all limbs were within the arm, while alternation was defined as three consecutive entries in three different arms (1, 2, 3 or 2, 3, 1, etc.). The percentage of alternation was calculated using the following formula: total alternation number/total number of entries–2) × 100.

### 4.4 Morris water maze

The apparatus used to perform the Morris water maze task consisted of a large circular pool (122 cm in diameter) with walls 76 cm high, which was filled with water maintained at 22° 2°C and, made opaque by the addition of a nontoxic white paint. Mice were given four daily trials for five consecutive days. Animals were trained to swim to a submerged Plexiglas platform starting each time from different positions. If they fail to find the platform within 60 s, they were manually guided to the platform and allowed to remain there for 15 s. Mice were trained to reach the hidden platform within 20 s (escape latency). On the fifth day, after 5 hr from the last training session, during the probe, mice were allowed to swim in the pool without the platform for 180 s, and the number of entries in the platform zone and the time spent in the different quadrants were recorded. During subsequent trials, the swimming speed was also determined (Lauretti, Li, et al., 2017).

### 4.5 Novel object recognition test

Mice were habituated to the open testing arena for two consecutive days (10 min each time). During the memory acquisition trial, each mouse was allowed to explore two identical objects for 10 min. For the memory retention phase, 1 hr after the memory acquisition trial, animals were exposed for 10 min to the presence of one familiar object and one novel object (a different shape and color). Object exploration time was recorded when the mouse touched the object directly with its nose, mouth, or forepaws. The discrimination index was calculated as the time spent near the new object divided by the cumulative time spent with both objects as previously described (Vagnozzi, Giannopoulos, & Praticò, 2017).

### 4.6 Electrophysiology studies

#### 4.6.1 Slice preparation

Mice from both groups were anesthetized with isoflurane and quickly decapitated, next brains were removed and hippocampi quickly dissected, placed in a tissue slicer (Stoelting Co. IL, USA), and 400 micron hippocampal sections cut. Slices were transferred into an incubation chamber filled with artificial cerebrospinal fluid (aCSF) of the following composition (in mM): 124 NaCl, 3 KCl, 2 MgCl_2_, 1.25 NaH_2_PO_4_, 2.5 CaCl_2_, 26 NaHCO_3_, 10 dextrose, and pH 7.4 adjusted with continuous bubbling of 95%O_2_/5%CO_2_ gas mixture. Slices were allowed to stay in the incubation chamber at least for 2 hr for recovery and then transferred to the experimental chamber for field potential recordings. Hippocampal slices from both male and female were used.

#### 4.6.2 Field potential recordings

Hippocampal slices were transferred to the submerged experimental chamber and continuously perfused with bubbled aCSF. All recordings were done at a temperature of 30–31°C controlled with PTC03 Temperature Controller (Scientific Systems Design Inc, USA). Field excitatory postsynaptic potentials (fEPSPs) were evoked by stimulation of Schaffer collaterals of hippocampus with 0.2 msec bi‐physic electrical pulse by platinum/iridium bipolar electrode (FHC, USA) connected to isolated pulse stimulator MODEL 2100 (A‐M systems, USA). Registration of fEPSPs was done in the *stratum radiatum* of CA1 area of hippocampus with a borosilicate glass electrode of 1.5–2 mOhm resistance pulled with PIP6 pipette puller (HEKA, USA) and filled with aCSF. The recording electrode was connected to a headstage of IE‐210 amplifier (Warner, USA). Recorded fEPSPs were filtered with LPF 202A low pass Bessel filter (Warner, USA) and digitized with Axon Digidata 1550b (Molecular Devices, USA). All data acquisition was done with Clampex 10.7 software from PClamp 10.7 software suit (Molecular Devices, USA) In order to study basal synaptic activity, the hippocampal slice was stimulated with current pulses of a different intensity and constant increment at 20 s inter‐pulse interval. Input–output curve was built as value of fEPSP slope vs amount of injected current. Next, short‐term and long‐term plasticity (STP and LTP) were assessed at intensity of stimulation needed to elicit fEPSP at 1/3 of a maximal response which was selected based on input–output curve analysis. STP was assessed with time dependency of paired‐pulse facilitation protocol. For this paired current pulses at different inter‐pulse intervals were used to elicit paired fEPSPs. Then ratio of fEPSP slope at pulse 2 to fEPSP slope at pulse 1 was built over respective inter‐pulse‐interval time points. In order to induce LTP, high‐frequency stimulation protocol was used. Before LTP induction, the baseline response to continuous stimulation was recorded for 20 min. Then, three consecutive tetanic pulses of 100 Hz and 1‐s duration at 60‐s inter‐pulse interval were applied in order to induce LTP. Values of fEPSP slope after LTP induction were normalized to the values of fEPSP slope at the baseline. All the data analysis for fEPSP slope assessment was performed by using the Clampfit 10.7 software from PClamp 10.7 software suit (Molecular Devices, USA).

#### 4.6.3 Immunoblot analyses

Immunoblot analyses were performed as previously described (Joshi et al., 2014). Briefly, proteins were extracted in enzyme immunoassay precipitation buffer containing 250 mM Tris base, 750 mM NaCl, 5% NP‐40, 25 mM EDTA, 2.5% sodium deoxycholate, 0.5% sodium dodecyl sulfate and an EDTA‐free protease and phosphatase inhibitors cocktail tablet (Roche Applied Science, Indianapolis, IN, USA), sonicated, centrifuged at 125,000 *g* for 45 min at 4°C, and supernatants used for immunoblot analysis. Total protein concentration was determined by using BCA Protein Assay Kit (Pierce, Rockford, IL, USA). Samples were electrophoretically separated using 10% Bis–Tris gels or 3%–8% Tris–acetate gel (Bio‐Rad, Richmond, CA, USA), according to the molecular weight of the target molecule, and then transferred onto nitrocellulose membranes (Bio‐Rad). They were blocked with Odyssey blocking buffer for 1 hr and then incubated with primary antibodies overnight at 4°C. After three washing cycles with T‐TBS, membranes were incubated with IRDye 800CW or IRDye 680CW‐labeled secondary antibodies (LI‐COR Bioscience, NE, USA) at 22°C for 1 hr. Signals were developed with Odyssey Infrared Imaging Systems (LI‐COR Bioscience). Actin and GAPDH were used as internal loading controls. Primary antibodies used in this paper are summarized in Table 2.

**Table 2:** Antibodies used in the study

Antibody	Catalog number	Company	Species
HT7	MN1000	Thermo	Mouse
AT8	MN1020	Thermo	Mouse
AT180	P10636	Thermo	Mouse
AT270	MN1050	Thermo	Mouse
PHF1	Gift	Dr. Peter Davis	Mouse
PHF13	9632	Cell Signaling	Mouse
T22	Gift	Dr. Kayez Rakez	Rabbit
cdk5	sc‐173	Santa Cruz	Rabbit
p35	sc‐820	Santa Cruz	Rabbit
P38	9212S	Cell Signaling	Rabbit
P‐p38 MAPK (T180Y182)	4511S	Cell Signaling	Rabbit
GSK 3b	sc‐9166	Santa Cruz	Rabbit
p‐GSK‐3beta (s9)	9336S	Cell Signaling	Rabbit
SNAP25	ab5666	Abcam	Rabbit
Syntaxin 1A	AF7237	RD SYSTEMS	Goat
Synaptobrein	AF4828	RD SYSTEMS	Goat
Complexin 1/2	AF7787	RD SYSTEM	Goat
SYP (Synaptophysin)	sc‐55507	Santa Cruz	Mouse
GluA1	ab31232	Abcam	Rabbit
GluA2	AB52180	Abcam	Rabbit
p‐Glua1 (S845)	PPS008	RD systems	Rabbit
p‐GluA1 (S831)	PPS007	RD systems	Rabbit
p‐GluA2 (S880)	ab52180	Abcam	Rabbit
PSD95	2507S	Cell Signaling	Rabbit
GFAP	sc‐33673	Santa Cruz	Mouse
Iba1	MABN92	Millipore	Mouse

#### 4.6.4 Formic acid extraction

Mouse brain homogenates were sequentially extracted in formic acid (FA) for the analysis of the insoluble protein fraction, as previously described (Lauretti & Praticò, 2017). Insoluble tau was immunoblotted with the HT‐7 antibody.

#### 4.6.5 Tau oligomers extraction

For the detection of tau oligomers, cortices were homogenized in PBS with protease and phosphatase inhibitors cocktail tablet (Roche Applied Science, Indianapolis, IN, USA, using a 1:3 dilution of tissue: PBS (w/v). Samples were centrifuged at 9,600 *g* for 10 min at 4°C, aliquoted and stored at −80°C until use (Lasagna‐Reeves et al., 2012). PBS‐soluble fractions were run on a 10% Bis–Tris gels (Bio‐Rad, Richmond, CA, USA), subsequently transferred onto nitrocellulose, blocked 1h at room temperature, and were probed overnight at 4°C with anti‐tau oligomer antibody T22 (1:1,000).

#### 4.6.6 Quantitative real‐time RT–PCR

RNA from mice brain tissues was extracted and purified using the RNeasy Mini Kit (Qiagen, Germantown, MD). As previously described (Vagnozzi et al., 2017), 1 μg of total RNA was used to synthesize cDNA in a 20 μl reaction using the RT2 First Strand Kit for reverse transcriptase–PCR (Super Array Bioscience). Human Tau gene was amplified by using the corresponding primer for human MAPT (#330001, Qiagen, Hilden, Germany). β‐Actin was used as an internal control gene to normalize for the amount of RNA. Quantitative real‐time RT–PCR was performed by using StepOnePlus Real‐Time PCR Systems (Applied Biosystems, Foster City, CA) and SYBR Green PCR Master Mix (Applied Biosystems, Foster City, CA). Each sample was run in triplicate, and analysis of relative gene expression was done by using the 2^−ΔΔCt^ method (Livak & Schmittgen, 2001).

## CONFLICTS OF INTEREST

The authors have no conflicting financial interest to disclose.

## AUTHOR’S CONTRIBUTIONS

EL and DP designed the study. EL, MN, and OD performed the experiments. EL and MN collected the data. EL, MN, and DP analyzed the data. EL, MN, and DP wrote the manuscript. All the authors have seen and approved the final version of the manuscript.

## Data Availability

Unpaired Student's *t* test (two‐sided) and one‐way ANOVA were performed using Prism 5.0 (GraphPad Software, La Jolla, CA, USA). All data are presented as mean ± SEM. Significance was set at *p* < .05.
